# Supplementary material for: ALDH3A1 Overexpression in Melanoma and Lung Tumors Drives Cancer Stem Cell Expansion, Impairing Immune Surveillance through Enhanced PD-L1 Output
Source: Cancers (Basel). 2019 Dec 6;11(12):1963. doi: 10.3390/cancers11121963 (PMC6966589; doi:10.3390/cancers11121963)
Supplement: Supplementary file 1 [file cancers-11-01963-s001.zip › Suppl.-final/cancers-642789-suppl-final.docx]

Article

ALDH3A1 Overexpression in Melanoma and Lung Tumor Drives Cancer Stem Cell Expansion, Impairing Immune Surveillance through Enhanced PD-L1 Output

Erika Terzuoli ^1^, Cristiana Bellan ^2^, Sara Aversa ^2^, Valerio Ciccone ^3^, Lucia Morbidelli ^3^, Antonio Giachetti ^3^, Sandra Donnini ^3,^* and Marina Ziche ^1,^*

**Figure S1.** ALDH3A1 expression in metastatic melanoma and NSCLC cells. (**a**) Expression of ALDH3A1 in WM ALDH3A1 overexpressing (3A1^high^) cells. (**b,c**) Expression of ALDH3A1 in WM ALDH3A1 (**b)** or HCC **(c)** ALDH3A1 KD (#1 and 2, 3A1^low^) compared to empty vector (Ctr). β-actin has been used to normalize loading. (**d,e**)**.** Recombinant ALDH3A1 activity in WM (**d**) HCC (**e**) Ctr (empty vector), #1 and #2(3A1^low^) cells maintained for 48 h in 10% FBS. Data are percentage NAPDH production over that of the control (means ±SEM, *n* = 6 from three independent experiments).

**Figure S2.** ALDH3A1 knock-down effects on cell viability and clonogenicity. (**a,b**) Cell viability in WM (**a**) HCC (**b**) Ctr, #1 and #2 (3A1^low^) cells exposed to complete medium with 0.1 or 10% FBS for 48 h. Cell survival values, reported as absorbance at 540 nm, were obtained by MTT assay. ***P<0.001 vs. Ctr cells. (**c,d**) Colony formation capability of WM (**c**) and HCC (**d**) Ctr, #1 and #2 (3A1^low^) cells exposed to complete medium with 1% FBS. Colonies (>75 cells) with 50% efficiency were counted. Results are expressed as number of colonies/well. *** *p* < 0.001, vs. Ctr cells.

**Figure S3.** ALDH3A1 modulation affects PGE-2 signaling. (**a–f**) COX-2 and mPGES1 mRNA expression in WM (Ctr, 3A1^high^) (**a**) WM **(c)** and HCC (**e**) (Ctr, 3A1^low^ #1) cells maintained in 10% FBS for 36 h. Data are reported as fold change vs. Ctr. *** *p* < 0.001 vs. Ctr. COX-2 and mPGES1 protein expression analysed by western blot in WM Ctr, 3A1^high^, MEL (**b**) or WMCtr, 3A1^low^ #1 and #2 (**d**) or HCC Ctr, 3A1^low^ #1 and #2 (**f**)**.**

**Figure S4.** ALDH3A1 affects cytokines important in immune-surveillance. (**a–d**) mRNA expression for (**a**,**c**) IL-4, IL-6, IL-8, IL-13 and TGFβ or (**b, d**) IL-12 and IFNγ in (**a** and **b**) WM Ctr, 3A1^low^#1 or 3A1^high^ cells and (**c** and **d**) HCC Ctr or 3A1^low^#1 cells maintained for 48 h in 10% FBS. Data are reported as fold change vs. Ctr cells *** *p* < 0.001; ** *p* < 0.01; * *p* < 0.05 vs. Ctr cells.

**Figure S5.** ALDH1A1 and retinoic acid signalling do not contribute to WM proliferation, clonogenicity and cytokine release in WM cells. (**a**) ALDH1A1 expression in WM cells evaluated by western blot analysis. Cells were maintained in 10% FBS for 48 h. β-actin has been used to normalize loading. (**b**) Proliferation of WM in absence/presence of ALDH1A1 enzymatic inhibitor CM037 (10 µM, 48 h). (**c**) Clonogenicity of WM treated or not with CM037 (10 µM) after 10 days of culture. (**d**) ALDH1A1 expression in tumorspheres cultured for three generations. Representative images of tumorspheres in presence/absence of CM037. Quantification of sphere number (** *p* < 0.01 vs. untreated cells) and tumorsphere area (*** *p*< 0.001 and * *p* < 0.05 vs. untreated cells). (**e**) Human cytokine ELISA Array in chemiluminescence in WM treated with CM037 (10 μM, 48 h). Data are expressed as percentage of fold change vs. Ctr cells. (**f**) PD-L1 analysis in WM Ctr, 3A1^low^, 3A1^high^after 48 h of culture (10 % FBS). PD-L1 expression in WM 3A1^high^ treated with RAR antagonist (AGN193109) and RXR antagonist (UVI 3003) for 48 h (each at 1 μM). β-actin was used as loading control.

**Table S1.** Panel of immunosuppressive cytokines in WM266-4 ALDH3A1^low^.

| **Immunosuppressive cytokines** | | | | |
| --- | --- | --- | --- | --- |
| **IL-13** | **IL-6** | **IL-10** | **IL-8** | **IL-4** |
| −36.46 | −23.35 | −6.58 | −31.77 | −20.59 |
| **IP10** | **TGFβ** | **RANTES** | **G-CSF** |  |
| **−**68.24 | **−**25.87 | **−**20.599 | **−**14.88 |  |

The release of cytokines was measured in medium of WM266-4 ALDH3A1^low^ (Sh#1) cells and Ctr cells maintained in 1% FBS for 48 h. Data are expressed as fold change of immunosuppressive cytokines in WM266-4 ALDH3A1^low^ (Sh#1) cells compared to Ctr cells (%). IL-13: interleukin-13, IL-6: interleukin-6, IL-10: interleukin-10, IL-8: interleukin-8, IL-4: interleukin-4, IP10: Interferon gamma-induced Protein 10, TGFβ: Transforming Growth Factor β, RANTES: Regulated on Activation, Normal T Cell Expressed and Secreted, G-CSF: Granulocyte-Colony Stimulating Factor.

**Table S2.** Panel of immunostimulatory cytokines in WM266-4 ALDH3A1^low^.

| **Immunostimulatory cytokines** | | | | |
| --- | --- | --- | --- | --- |
| **IFNγ** | **IL-12** | **IL-1A** | **IL-17a** | **IL-2** |
| **18.36** | **50.23** | **50.93** | **24.9** | 68.24 |
|  | **GM-CSF** | **MPI-1α** | **SCF** |  |
|  | 66.68 | 31.77 | 66.68 |  |

The release of cytokines was measured in medium of WM266-4 ALDH3A1^low^ (Sh#1) cells and. Ctr cells maintained in 1% FBS for 48 h. Dara are expressed as fold change of immunostimulatory cytokines in WM266-4 ALDH3A1^low^ (Sh#1) cells compared to Ctr cells (%). IFNγ: interferon γ, IL-12: interleukin-12, IL-1A: interleukin-1A, IL-17a: interleukin-17a, IL-2: interleukin-2, GM-CSF: Granulocyte-Macrophage Colony-Stimulating Factor, MIP-1α: Macrophage Inflammatory Protein-1α, SCF: Stem Cell Factor.

**Table S3.** ALDH3A1 overexpression inhibits proliferation of CFSE-stained PBMC.

| **Cells** | **MFI** | **CFSE % LOW** | **CFSE % HIGH** |
| --- | --- | --- | --- |
| PBMC | 929 | 0 | 100 |
| PBMC+ anti CD3/CD28 | 329 | 10.9 | 89.1 |
| PBMC+ anti CD3/CD28 + MC HCC4006 Ctr | 173 | 22.6 | 77.4 |
| PBMC+ anti CD3/CD28 + MC HCC4006 ALDH3A1^low^ | 57 | 43.9 | 56.1 |
| PBMC+ anti CD3/CD28 +MC WM266-4 high ALDH3A1 | 324 | 15.2 | 84.8 |
| PBMC+ anti CD3/CD28 +MC WM266-4 low ALDH3A1 | 167 | 24.9 | 75.1 |

Proliferation of CFSE-stained PBMC cultured in conditioned media from tumor cells. Mean Fluorescence Unit (MFI) and % of CFSE low and high cells are reported.

**Table S4.** Clinical-pathological characteristics in lung cancer.

| **Tumor** | **Gender** | **Age** | **Stage** | **Grade** | **ALDH3A1** | | **COX-2** | | **PD-L1** |
| --- | --- | --- | --- | --- | --- | --- | --- | --- | --- |
|  |  |  |  |  | **Positive cells %** | **Intensity** | **Positive cells %** | **Intensity** | **Positive cells %** |
| Lung | Male | 51 | pT1CN0MX | G2 | 30% | +++ | <5% | + | 80 |
| Lung | Female | 85 | pT1cN0MX | G2 | 90% | +++ | 90% | ++ | 90 |
| Lung | Male | 63 | pT3N0MX | G2 | 90% | +++ | 90% | +++ | 75 |
| Lung | Male | 81 | pT1cN1MX | G2 | 90% | +++ | 70–80% | ++ | 90 |
| Lung | Male | 75 | pT1bN0MX | G2 | 90% | +++ | 90% | + | 55 |
| Lung | Female | 72 | pT1cN0MX |  | 70% | +++ | 70–80% | + | 80 |
| Lung | Male | 70 | pT1bN0Mx | G2 | 90-100% | +++ | 90% | ++ | 10 |
| Lung | Male | 69 | pT1aNXMX | G2 |  |  |  |  |  |
| Lung | Female | 77 |  |  | 60% | + |  |  |  |
| Lung | Male | 74 | pTiapN0 | G1 | 70% | ++ | neg | neg | neg |
| Lung | Male | 59 | pT1cpN0G1 | G1 | 80% | ++ | neg | neg | 10 |
| Lung | Male | 70 | pT1bN0MX | G2 |  |  | 20% | ++ | neg |
| Lung | Male | 74 | pT1cN1MX | G3 | 30% | +++ | 10% | ++ | 60 |

Clinical-pathological characteristics in tissue samples from patients with pulmonary adenocarcinoma collected from the University Hospital of Siena after written informed consent.

**Table S5.** Clinical-pathological characteristics in melanoma.

| **Tumor** | **Gender** | **Age** | **Stage** | **Grade** | **ALDH3A1** | | **COX-2** | | **PD-L1** |
| --- | --- | --- | --- | --- | --- | --- | --- | --- | --- |
|  |  |  |  |  | **Positive cells %** | **Intensity** | **Positive cells %** | **Intensity** | **Positive cells %** |
| melanoma | Female | 86 | pT4bNxMx | Nd | 70 | + | 50% | ++ | 10 |
| melanoma | Female | 70 | no stage | Nd | 100% | ++ |  |  | <5 |
| melanoma | Male | 78 | pT3aNxMx | Nd | 90% | ++ | 90% | ++/+++ | 5 |
| melanoma | Male | 69 | pT2bNxMx | Nd | 90% | + | 40% | + | 20 |
| melanoma | Male | 83 | Metastaticlymphnode | Nd | 80% | ++/+++ | 50% | + | 0 |
| melanoma | Male | 58 | pT1bNxMx | Nd | 90% | +++ | 90% | +++ | 5 |
| melanoma | Female | 27 | pT1bNxMx | Nd | 80% | +++ | 40% | ++ | <5 |

Clinical-pathological characteristics in tissue samples from patients with skin melanoma collected from the University Hospital of Siena after written informed consent.

**Table S6.** List of qPCR primers. Sequences (5’-3’) of forward and reverse primers used in the manuscript.

| **NAME** | **Forwardprimer (5’-3’)** | **Reverse primer (5’-3’)** |
| --- | --- | --- |
| *PTGS2* | GCTTTATGCTGAAGCCCTATGA | TCCAACTCTGCAGACATTTCC |
| *PTGES1* | GGGACTTGATGTTCCTTCCA | ATACACACACACGGGCACAC |
| *OCT4* | ACATCAAAGCTCTGCAGCAAAGAACT | CTGAATACCTTCCCAAATAGAACCC |
| *SOX2* | TTGCTGCCTCTTTAAGACTAGGA | TAAGCCTGGGGCTCAAACT |
| *NANOG* | ACATGCAACCTGAAGACGTGTG | CATGGAAACCAGAACACGTGG |
| *TWIST* | AGCTACGCCTTCTCGGTCT | CCTTCTCTGGAAACAATGACATC |
| *IL-4* | CCTTCTCTGGAAACAATGACATCT | GGTTTCCTTCTCAGTTGTGTTCTT |
| *IL-6* | GATGAGTACAAAAGTCCTGATCCA | CTGCAGCCACTGGTTCTGT |
| *IL-8* | GAGCACTCCATSSGGCACAAA | ATGGTTCCTTCCGGTGGT |
| *IL-12* | TCACCCCCACCTCTCTAAAA | TTTGCCTTCCAGACACTTACG |
| *IL-13* | AGCCCTCAGGGAGCTCAT | TGATGCTCCATACCATGGCTG |
| *IFNγ* | GGCATTTTGAAGAATTGGAAAG | TTTGGATGCTCTGGTCATCTT |
| *TGFβ* | AGCAGCACGTGGAGCTGT | CAGCCGGTTGCTGAGGTA |
| *CHD1* | CCAGAAACGGAGGCCTGAT | AGAACGCATTGCCACATACACT |
| *Zeb1* | GATGATGAATGCGAGTCAGATGC | CTGGTCCTCTTCAGGTGCC |
| *VIM* | AGTCCACTGAGTACCGGAGAC | CATTTCACGCATCTGGCGTTC |
| *FN1* | GAACTATGATGCCGACCAGAA | GGTTGTGCAGATTTCCTCGT |
| *RPL19* | GATGCCGGAAAAACACCTTG | TGGCTGTACCCTTCCGCTT |
