# Supplementary material for: ALDH3A1 Overexpression in Melanoma and Lung Tumors Drives Cancer Stem Cell Expansion, Impairing Immune Surveillance through Enhanced PD-L1 Output
Source: Cancers (Basel). 2019 Dec 6;11(12):1963. doi: 10.3390/cancers11121963 (PMC6966589; doi:10.3390/cancers11121963)

Figure 1

(a)

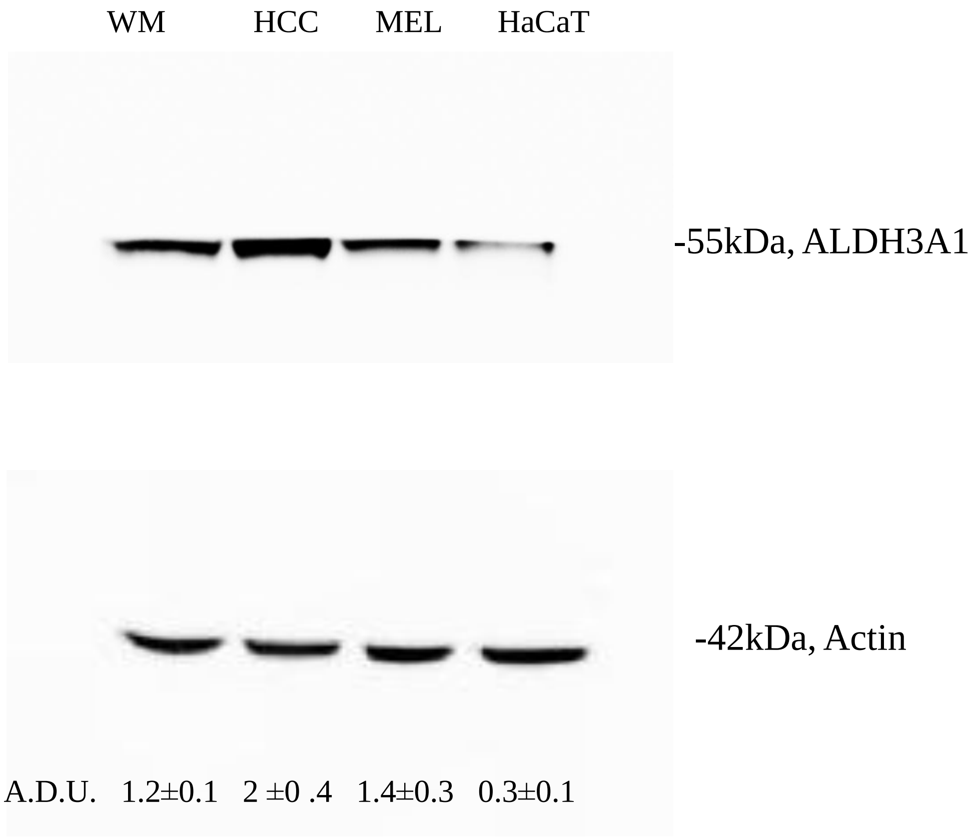
WM
HCC
MEL
HaCaT
-55kDa, ALDH3A1
-42kDa, Actin
A.D.U.
1.2±0.1
2 ±0 .4
1.4±0.3
0.3±0.1

Figure 2

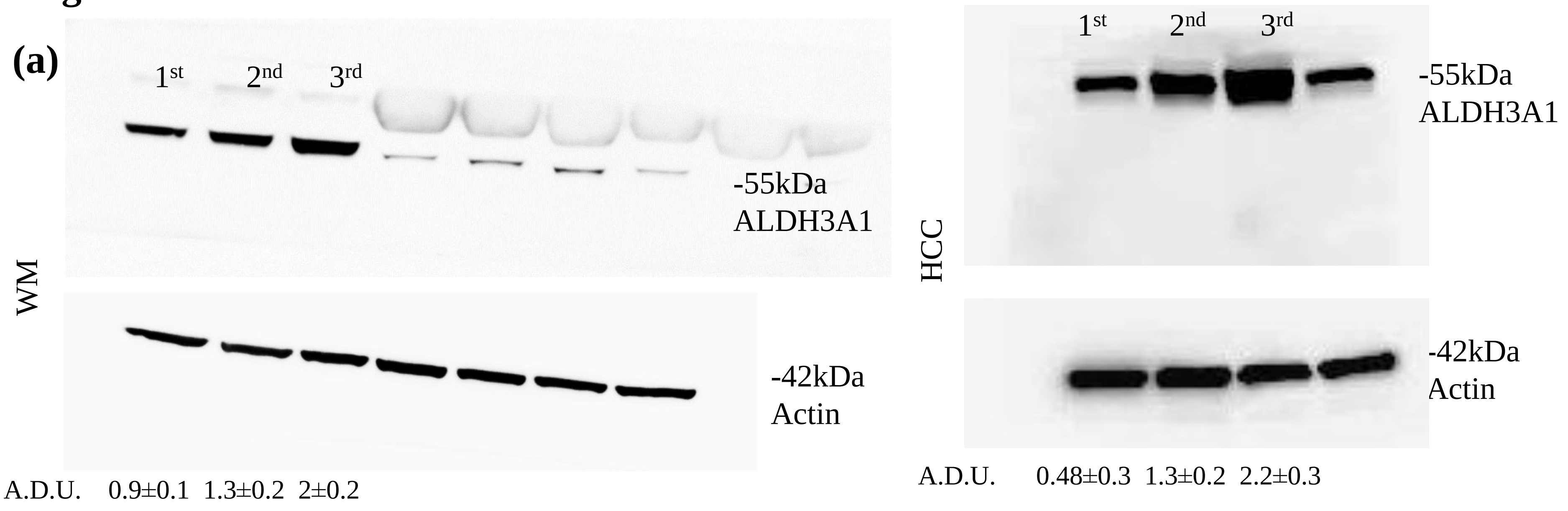

(a)
WM
1st
2nd
3rd
-55kDa
ALDH3A1
-42kDa
Actin
A.D.U.
0.9±0.1
1.3±0.2
2±0.2
HCC
1st
2nd
3rd
-55kDa
ALDH3A1
-42kDa
Actin
A.D.U.
0.48±0.3
1.3±0.2
2.2±0.3

Figure 2

(b)

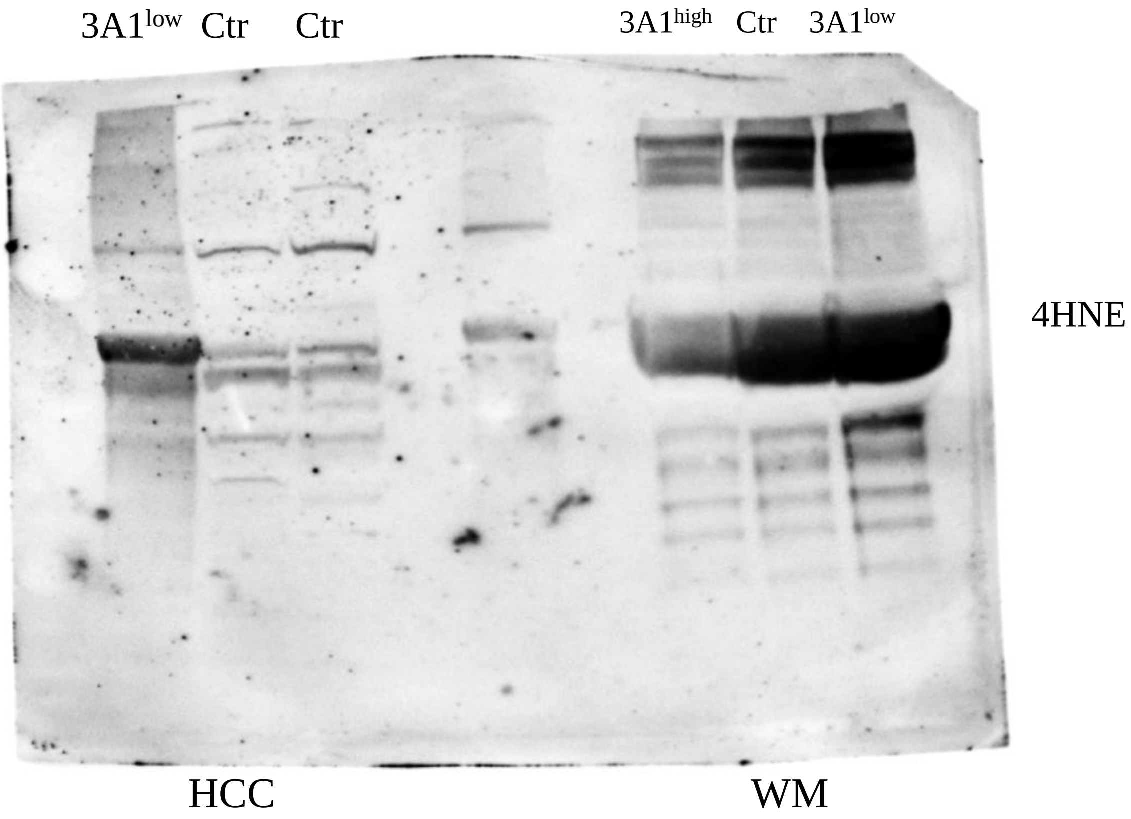

Figure 2

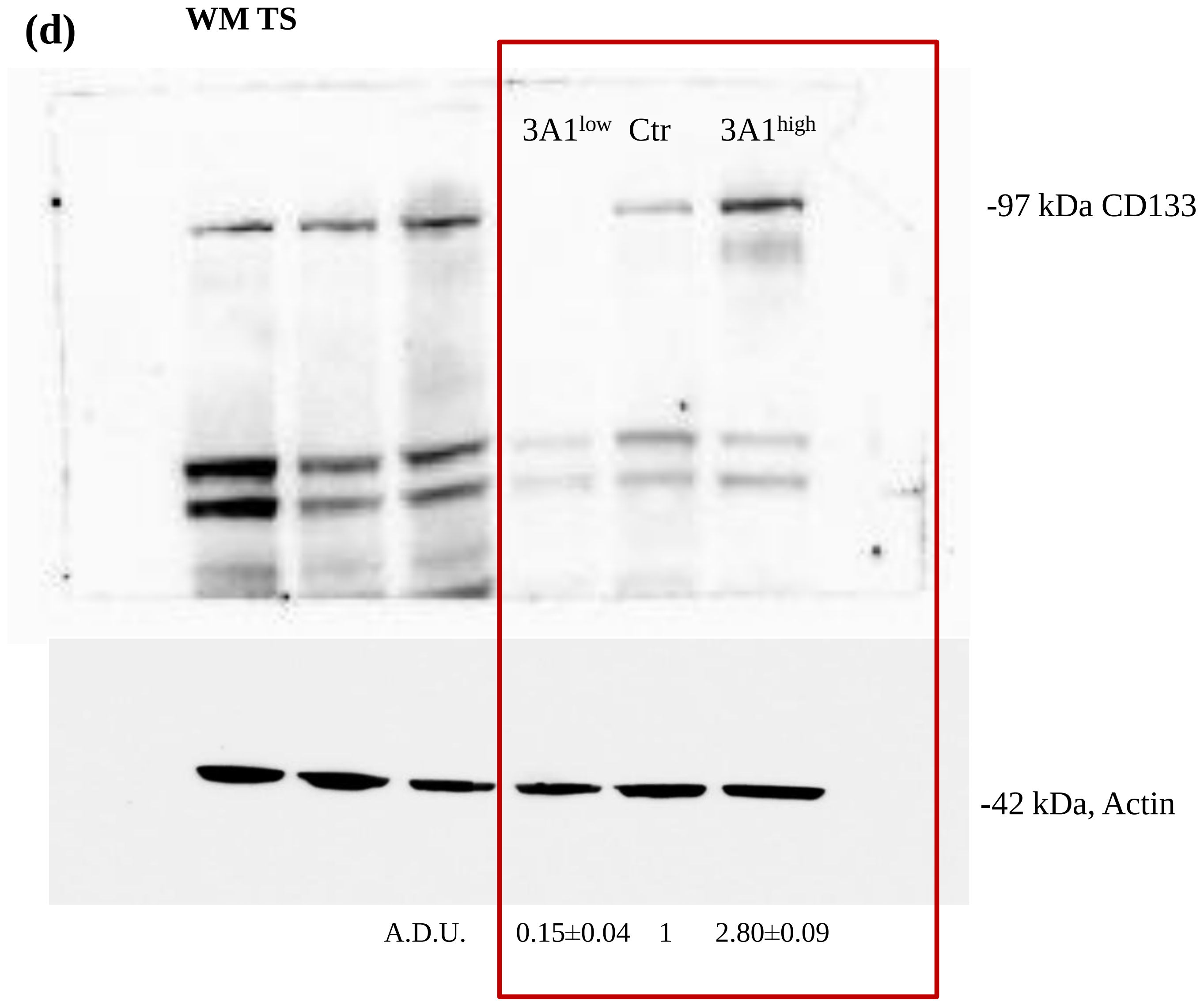
(d)
WM TS
3A1low Ctr 3A1high
-97 kDa CD133
-42 kDa, Actin
A.D.U. 0.15±0.04 1 2.80±0.09

Figure 2
(d)

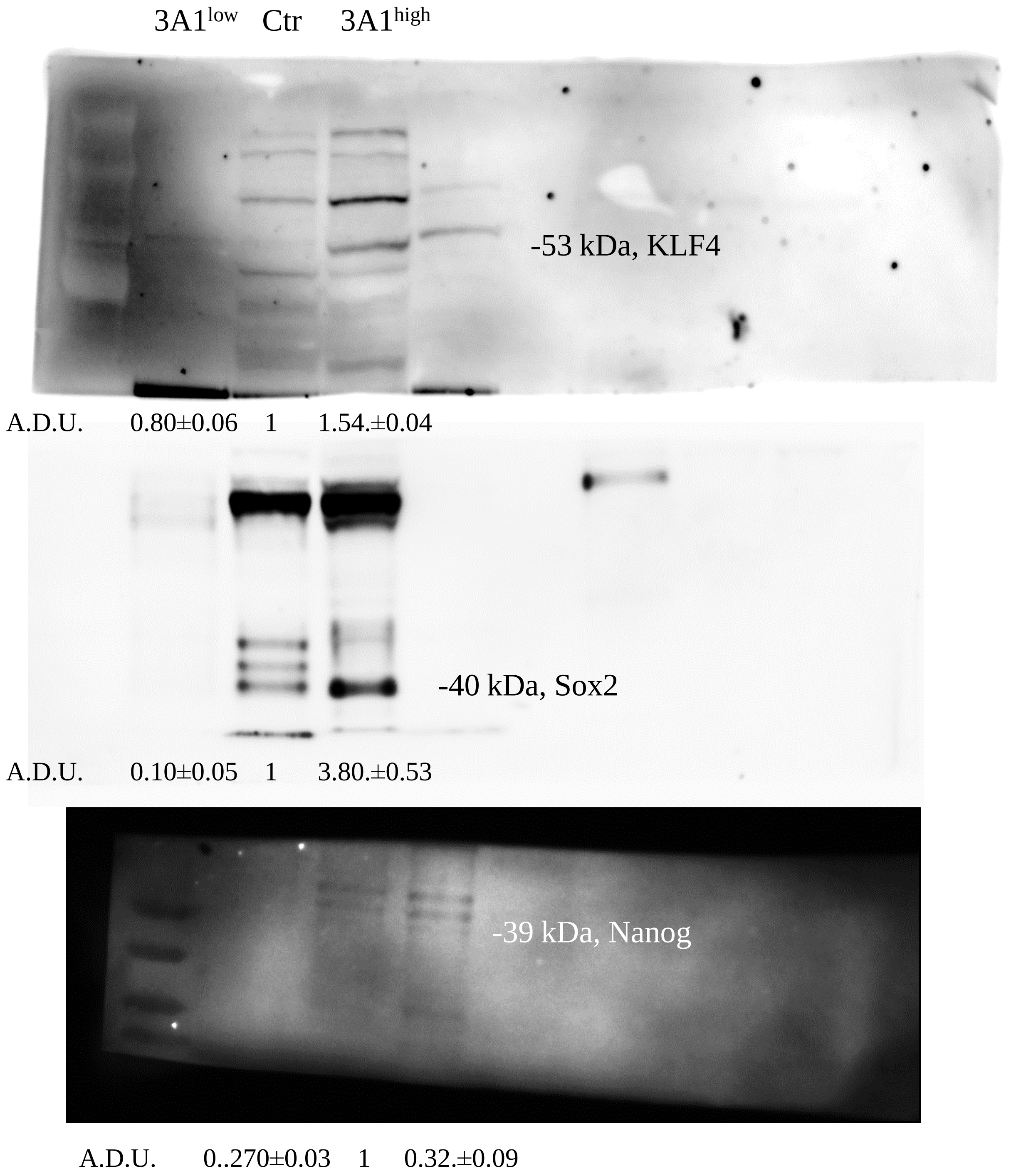
3A1low Ctr 3A1high
-53 kDa, KLF4
A.D.U. 0.80±0.06 1 1.54.±0.04
-40 kDa, Sox2
A.D.U. 0.10±0.05 1 3.80.±0.53
-39 kDa, Nanog
A.D.U. 0..270±0.03 1 0.32.±0.09

Figure 2

(d)

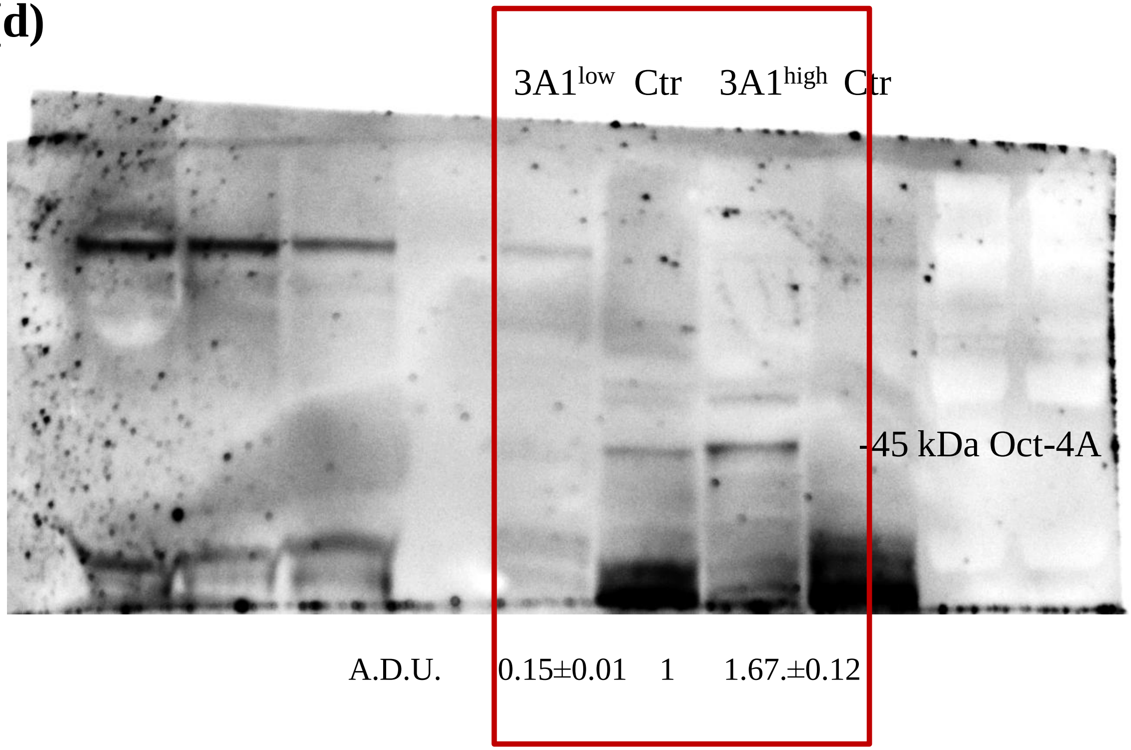

Figure 2

(f)

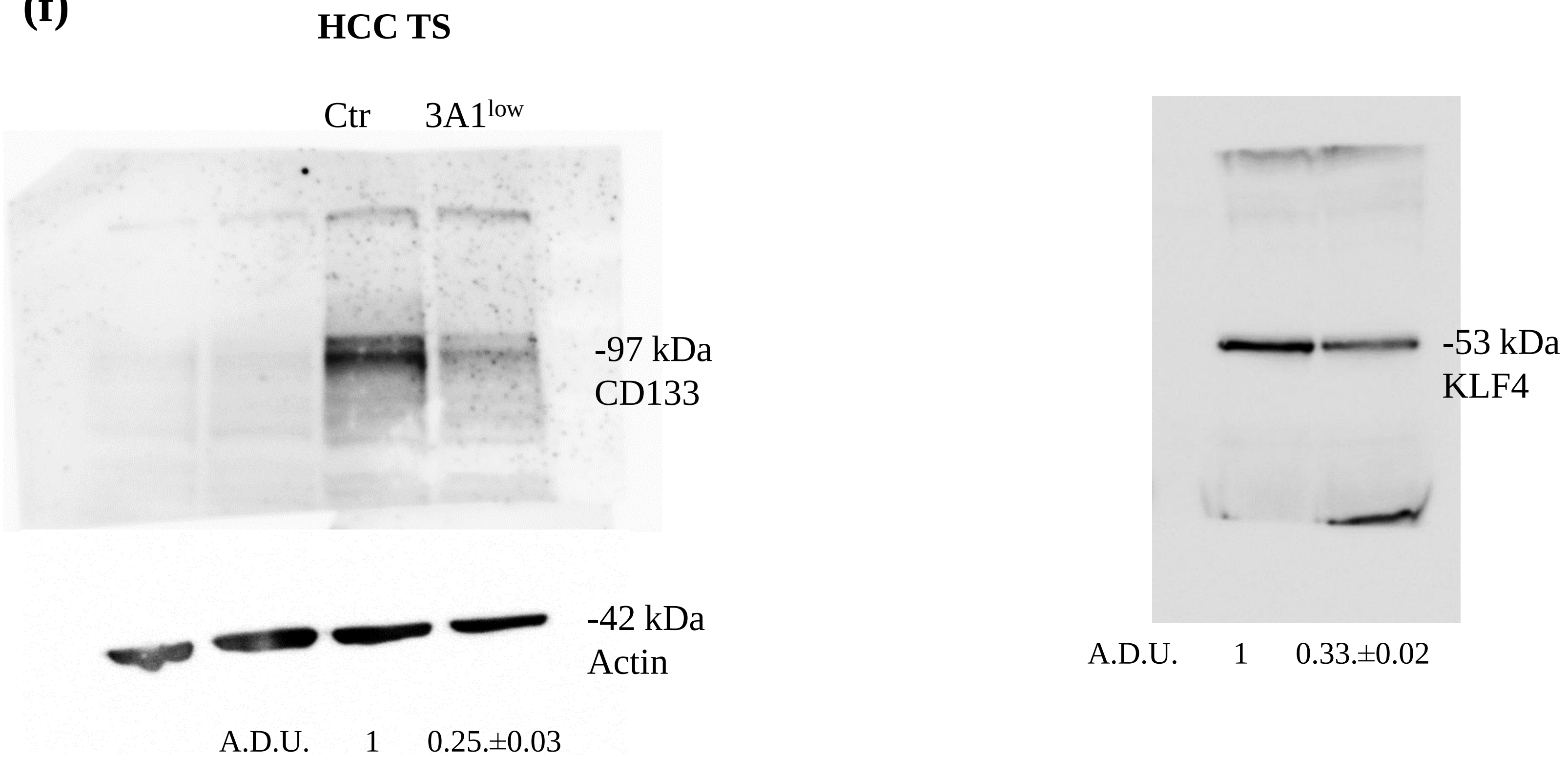
HCC TS
Ctr
3A1low
-97 kDa
CD133
-42 kDa
Actin
A.D.U. 1 0.25.±0.03
-53 kDa
KLF4
A.D.U. 1 0.33.±0.02

Figure 2

(f)

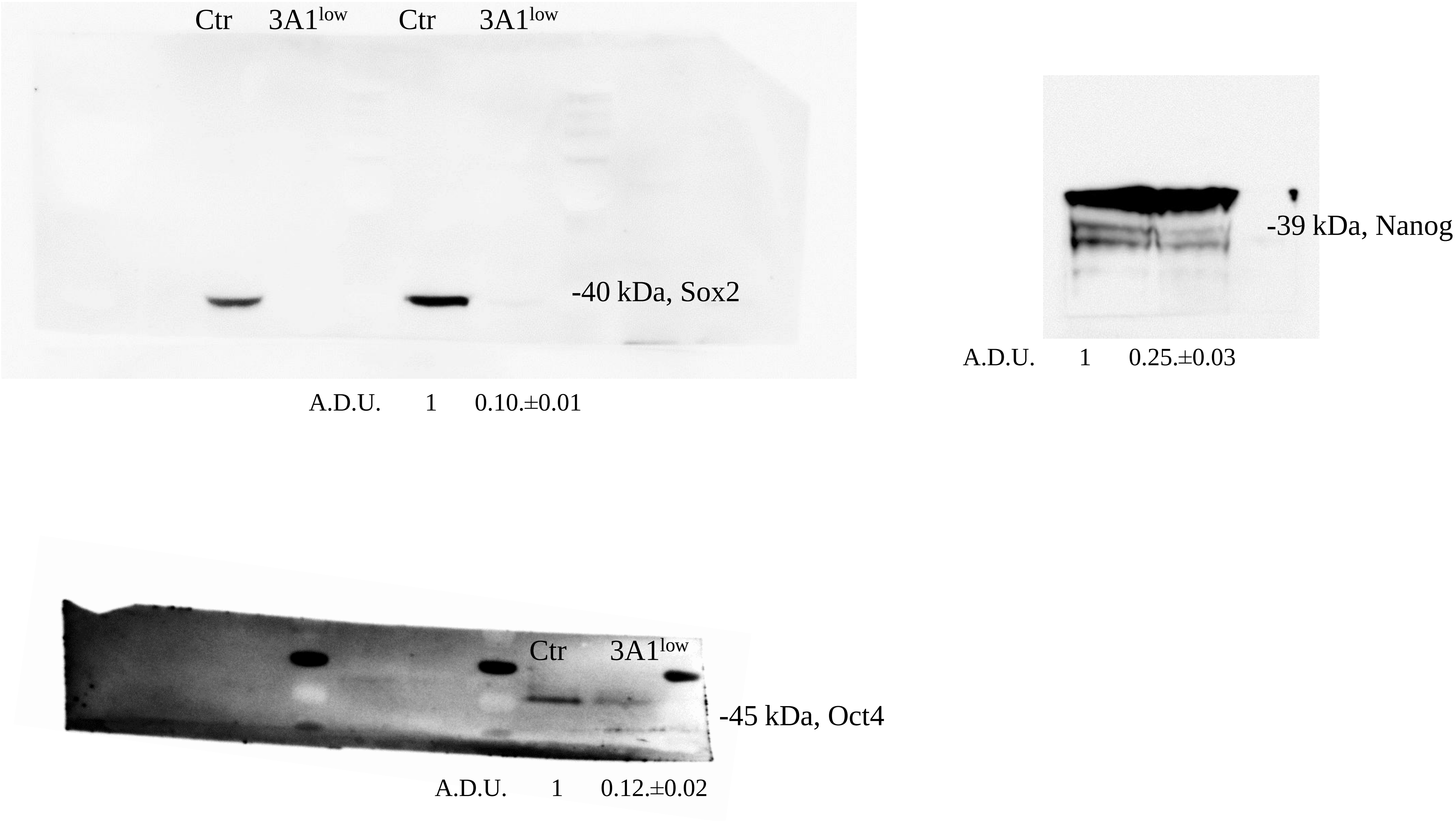
Ctr
3A1low
Ctr
3A1low
-40 kDa, Sox2
A.D.U.
1
0.10.±0.01
-39 kDa, Nanog
A.D.U.
1
0.25.±0.03
Ctr
3A1low
-45 kDa, Oct4
A.D.U.
1
0.12.±0.02

Figure 6

(a)

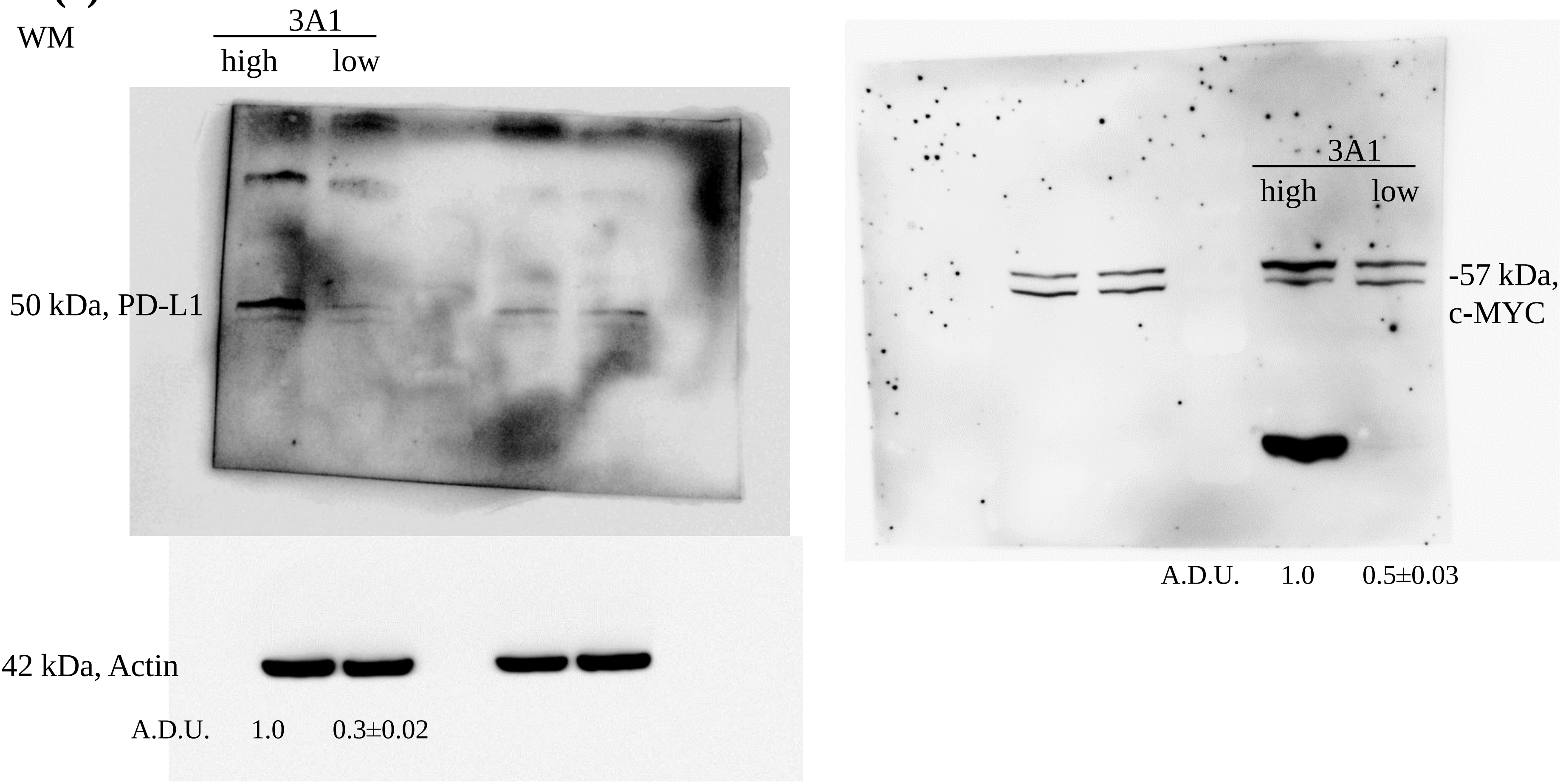

WM
3A1
high
low
50 kDa, PD-L1
42 kDa, Actin
A.D.U.
1.0
0.3±0.02
3A1
high
low
-57 kDa,
c-MYC
A.D.U.
1.0
0.5±0.03

(b)

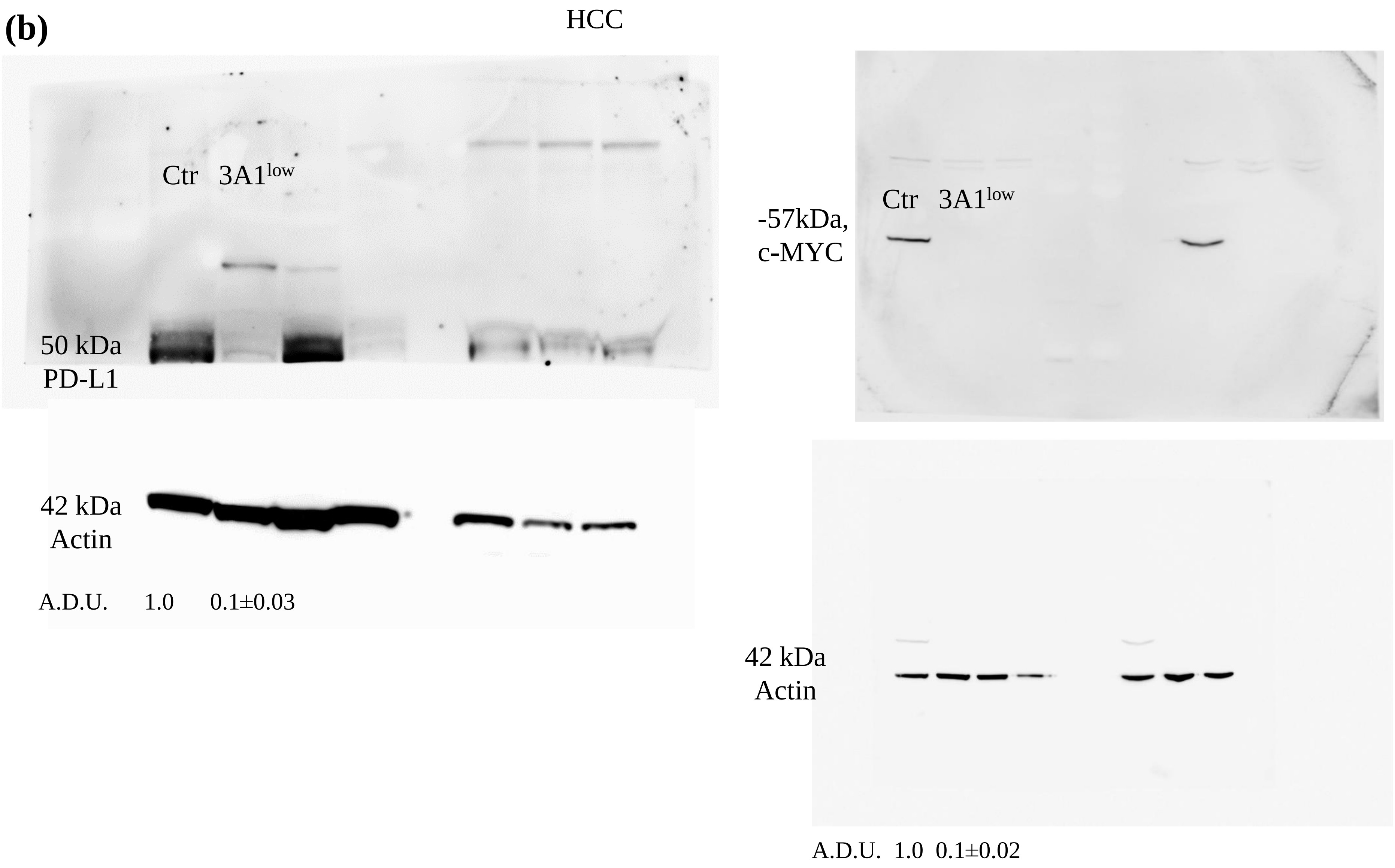
HCC
Ctr 3A1low
50 kDa
PD-L1
42 kDa
Actin
A.D.U. 1.0 0.1±0.03
Ctr 3A1low
-57kDa,
c-MYC
42 kDa
Actin
A.D.U. 1.0 0.1±0.02

SUPPLEMENTARY MATERIAL

Supplementary Fig. 1

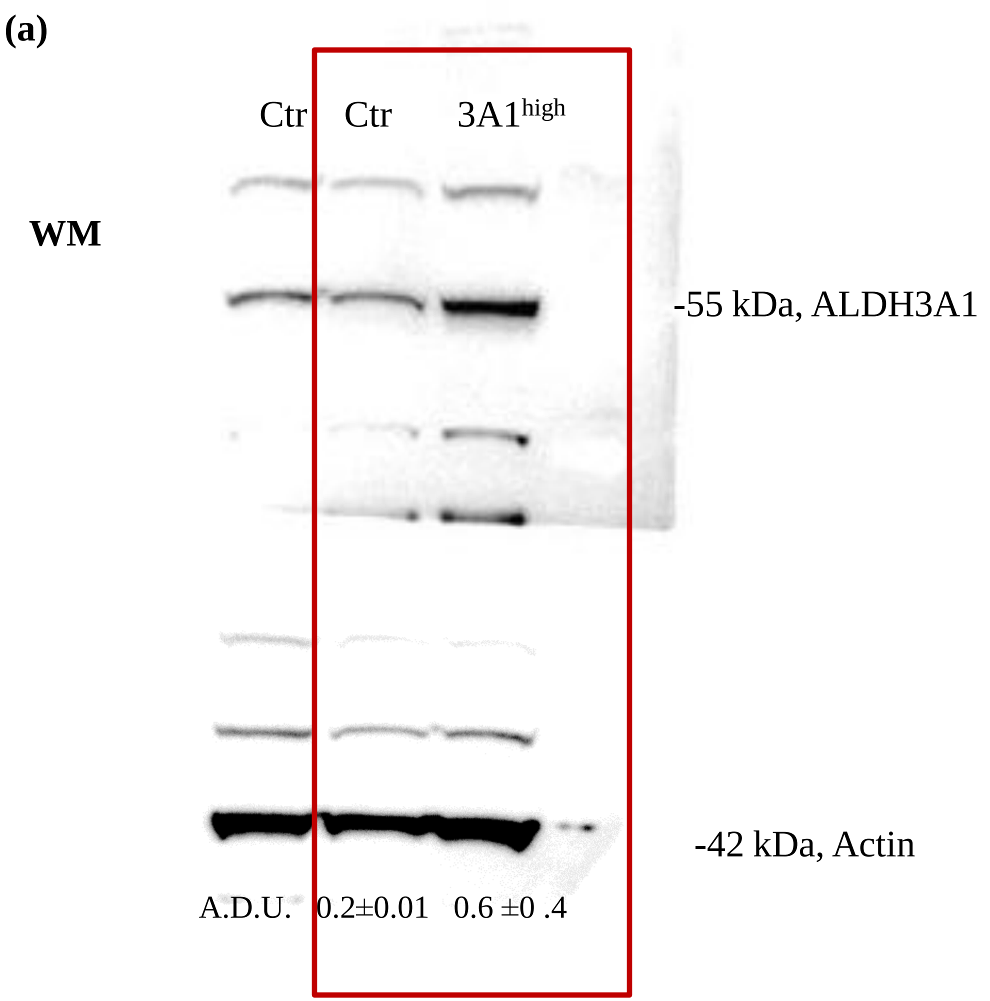

Supplementary Fig. 1 **(b)**

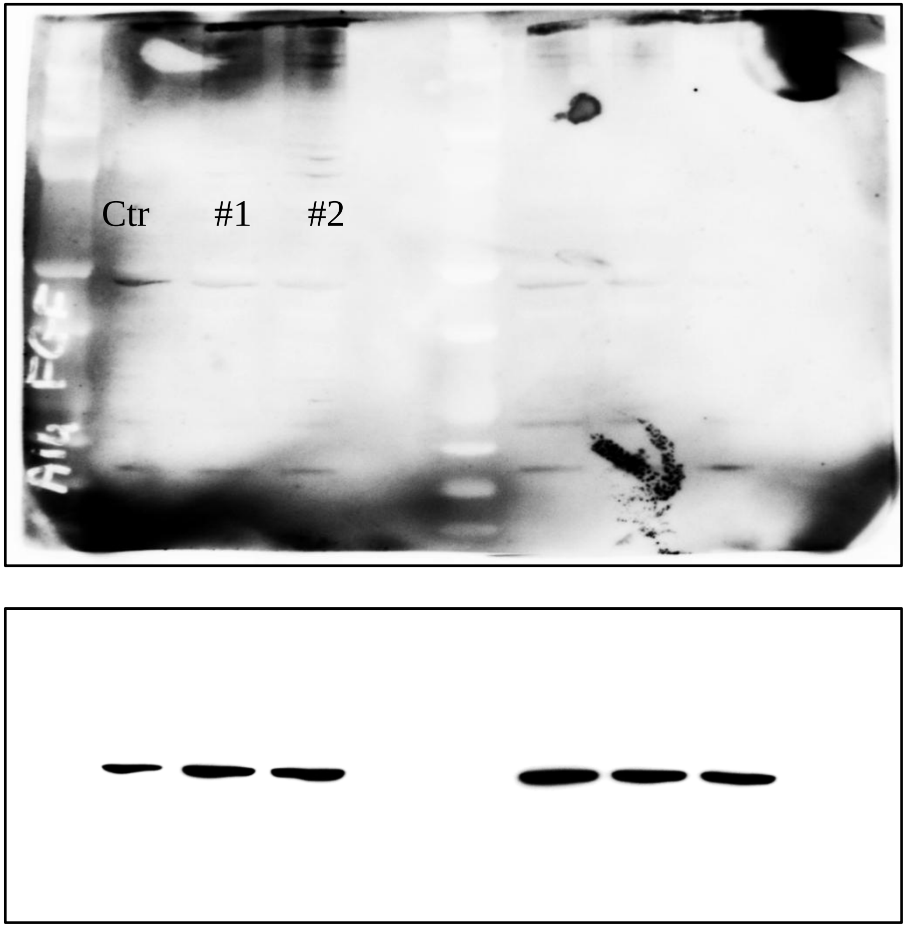

WM

-55 kDa, ALDH3A1

-42 kDa, Actin

A.D.U.	0.2± 0.01	0.05 ± 0.04	0.03± 0.01

Supplementary Fig. 2 (c)

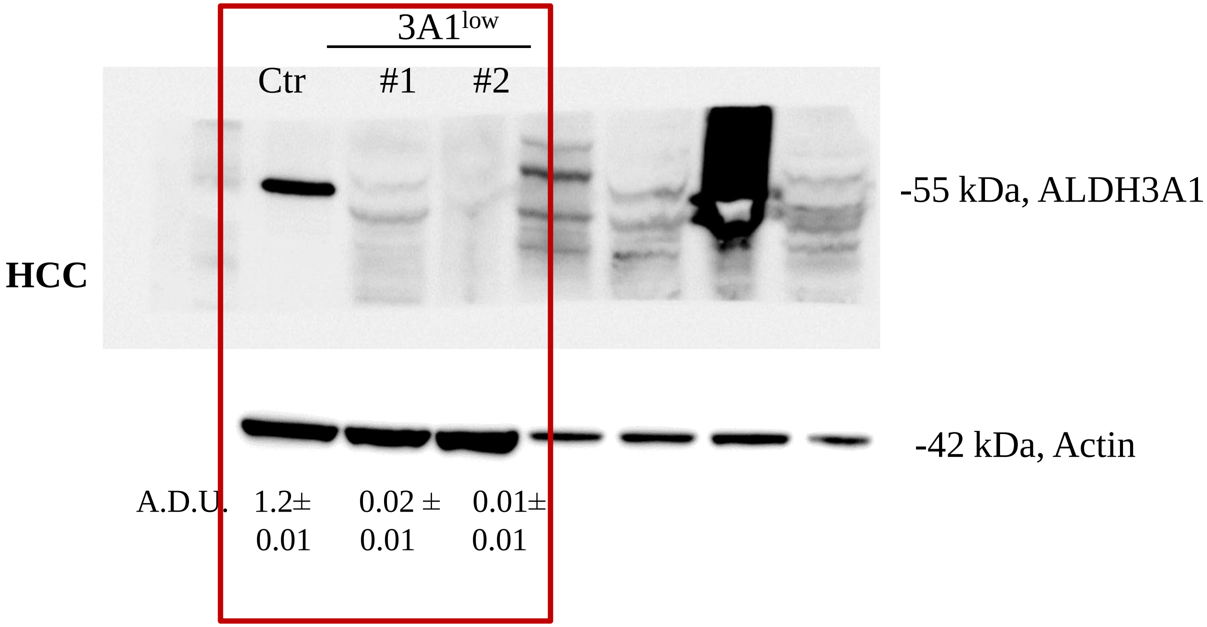

Supplementary Fig. 3

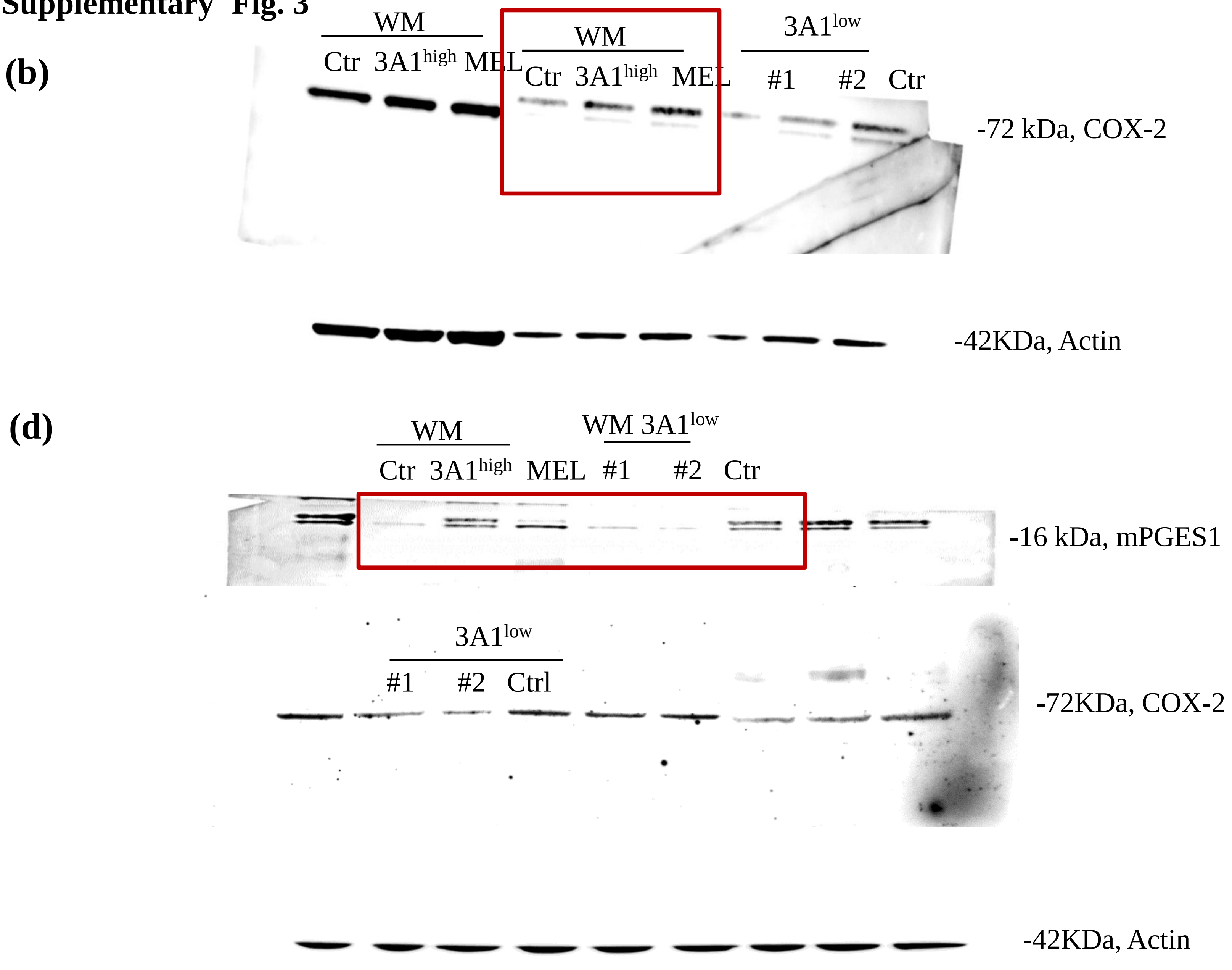

Supplementary Fig. 3

(f)

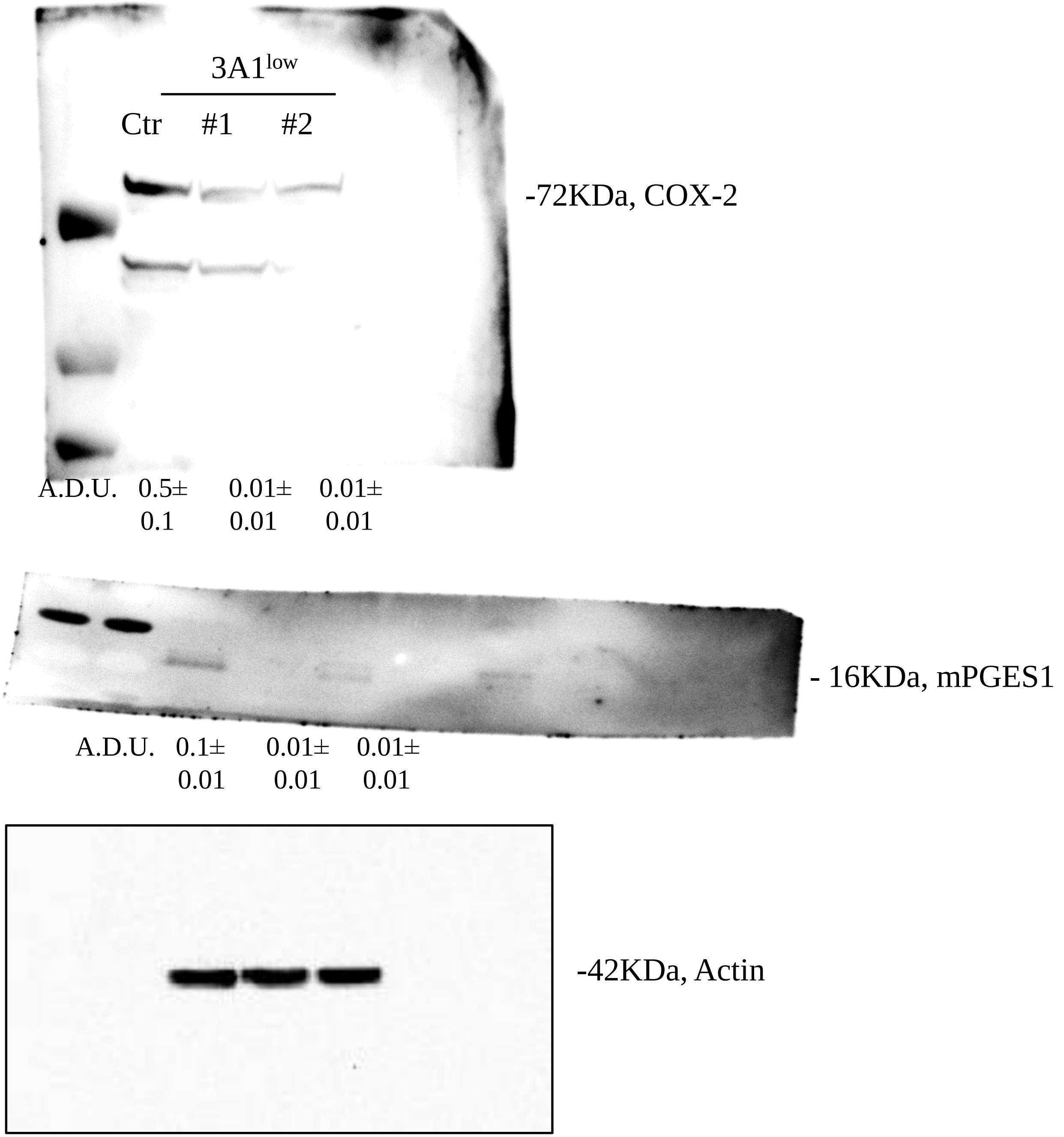

Supplementary Fig. 3

(g)

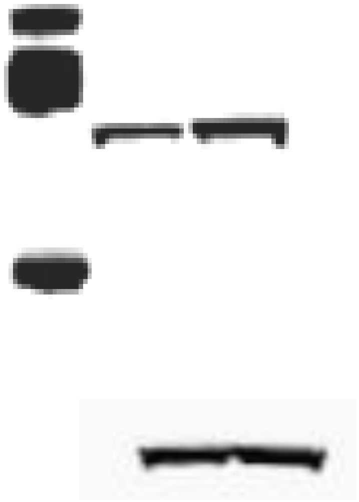

Supplementary Fig. 5

(a)

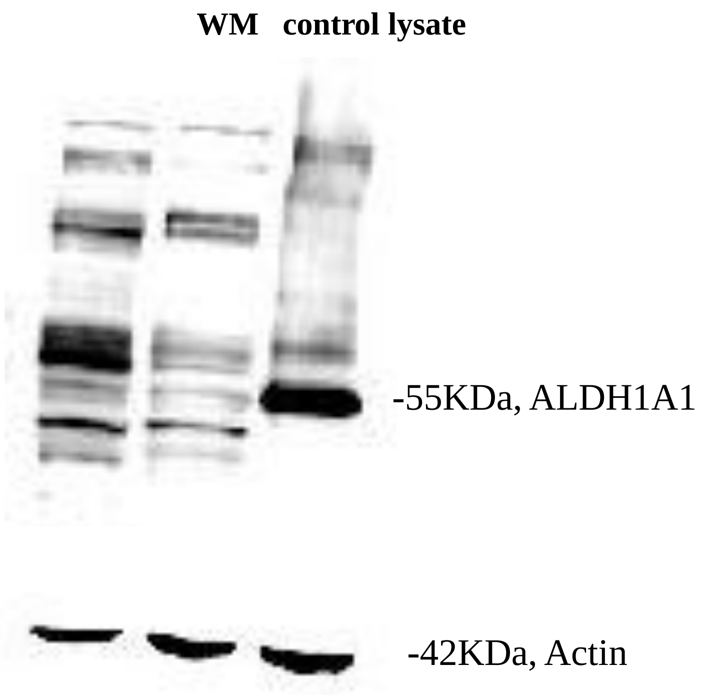

Supplementary Fig. 5

(d)

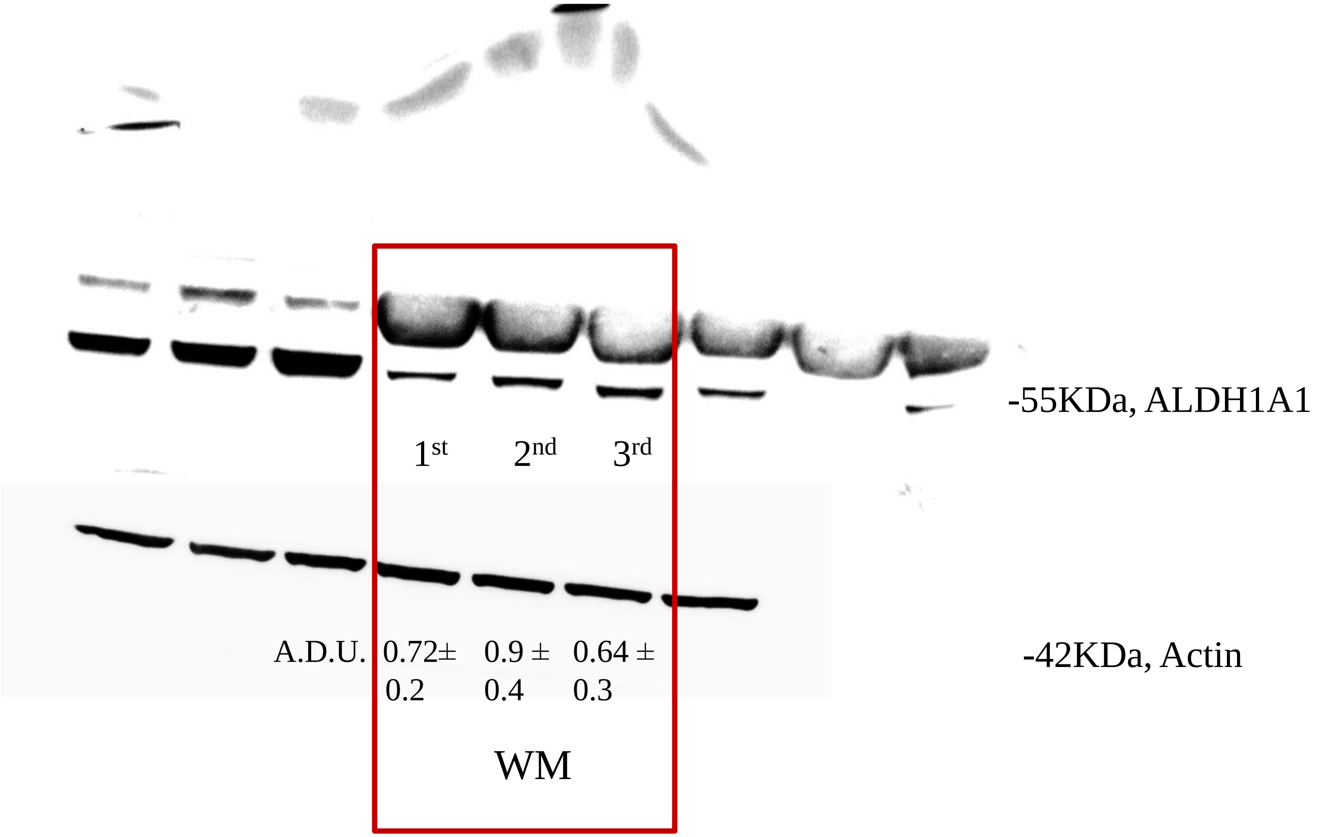

Supplementary Fig. 5

f

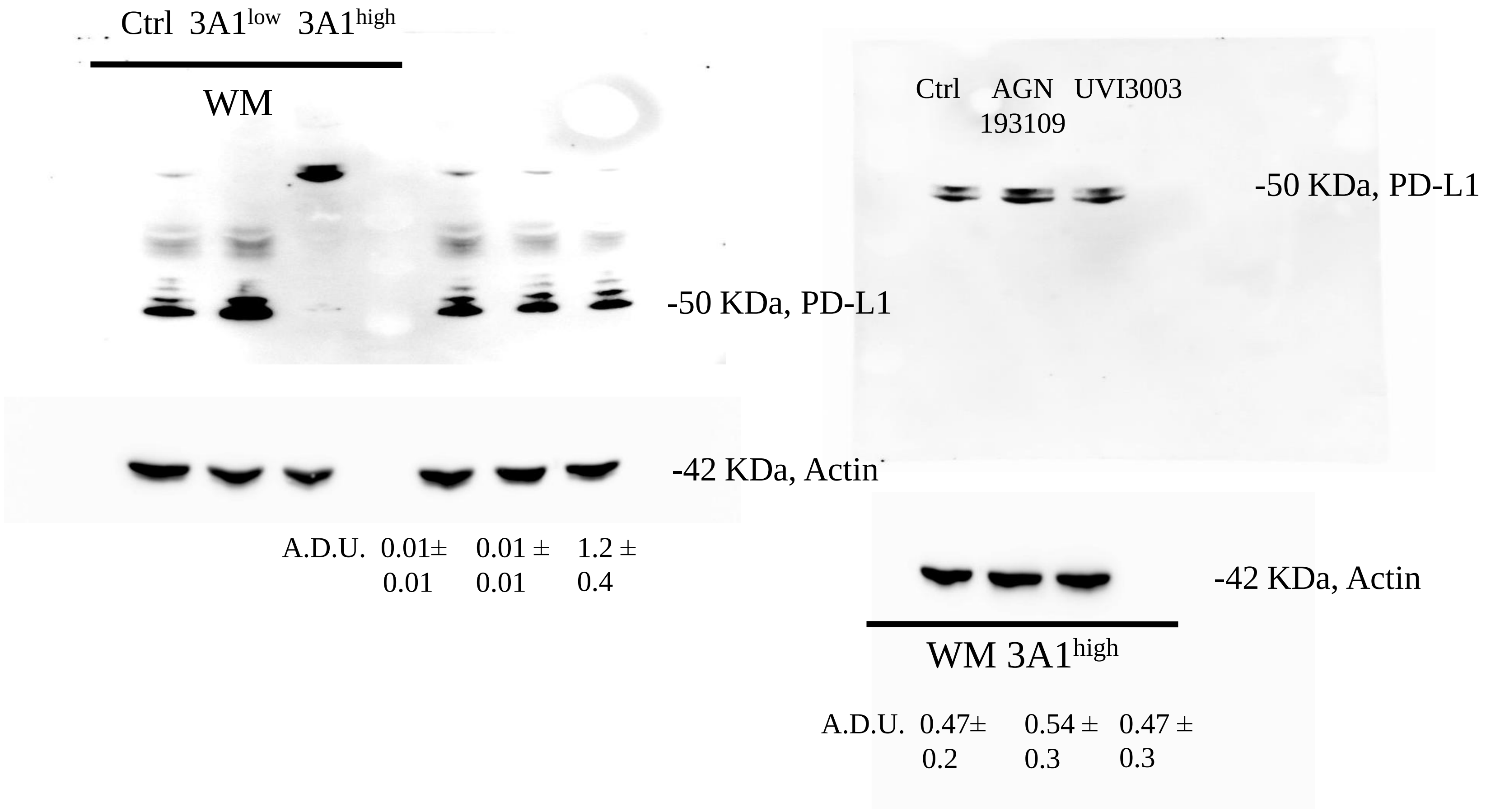

Supplement: Supplementary file 1 [file cancers-11-01963-s001.zip › Suppl.-final/cancers-642789-western blot-R1.pdf]
